# 

**Published:** 1907-03-23

**Authors:** 


### IMPORTANT NOTICE.

**On account of the Easter Holidays, we beg to announce that we shall go to press with our issue of the 30th MARCH on TUESDAY NIGHT, the 26th inst. Advertisements intended for insertion in this issue should therefore reach us by 5 p.m. ON TUESDAY, the 26th inst.**

### MUSCLES AND NERVES

**An Atlas of the Superficial Muscles and the Principal Motor Nerves of the Human Body for the use of Students of Anatomy and Nurses.**

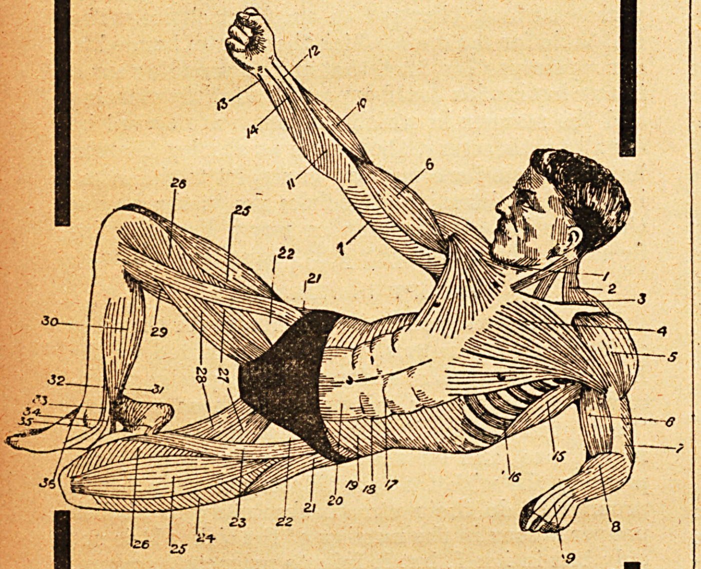

By LOUIS B. RAWLING, F.R.C.S.,

*Assistant Surgeon and Senior Demonstrator of Anatomy, St. Bartholomew's Hospital.*

**Price 3/6 net, 3/9 post free.**

This Atlas contains a series of photographs of a well-developed male in such positions calculated to show off the superficial muscles to the best advantage. Opposite to each figure is a plan which not only illustrates the arrangement and direction of the muscles called into action, but also indicates the situation of the more important motor nerves.

The work cannot fail to be of the greatest practical utility to all Nurses and those who desire to gain a thorough insight into the superficial muscular formation of the human body, and obtain a general idea as to the position of the nerves which supply those muscles.

*Of all Booksellers, and of*

THE SCIENTIFIC PRESS, Limited,
28 & 29 SOUTHAMPTON STREET, STRAND, LONDON, W.C.

### 1/6 NURSING HANDBOOKS.

By Post, 1/8

**SURGICAL INSTRUMENTS AND APPLIANCES USED IN OPERATIONS.**

An Illustrated and Classified List, with Explanatory Notes.

By Harold Burrows, M.B.Lond., B.S., F.R.C.S.

New Edition, Revised and Enlarged.

**ART OF FEEDING THE INVALID.**

By a Medical Practitioner and a Lady Professor of Cookery.

New and Popular Edition, with Special Chapter on Diet for Consumptives.

**THE CARE OF CHILDREN.**

Practical Hints for Mothers and Nurses at Home and Abroad.

By Robt. J. Blackham, D.P.H.Lond. Revised and Enlarged Edition.

**LECTURES ON HOME NURSING FOR THE POOR.**

By a District Nurse. Illustrated.

**OUTLINES OF ROUTINE IN DISTRICT NURSING.**

By M. Loane, Superintendent of District Nurses, Portsmouth.

1/9 post free.

**OUTLINES OF INSANITY.**

A Popular Treatise on the Salient Features of Insanity.

By Francis H. Walmsley, M.D.

Popular Edition.

**NURSING OF TROPICAL FEVERS.**

By S. F. Pollard, late Sister, Army Nursing Reserve.

The Work is based upon the Personal Experience of the Author Abroad.

*Complete Catalogue post free on application.*

THE SCIENTIFIC PRESS, Ltd.,
Nursing Publishers and Booksellers,
28 & 29 SOUTHAMPTON STREET, STRAND, LONDON, W.C.

